# Diffuse Alveolar Hemorrhage Mimicking Pneumonia in a Patient With Interstitial Lung Disease on Ticagrelor

**DOI:** 10.7759/cureus.107651

**Published:** 2026-04-24

**Authors:** Muhammad Usman Shahbaz, Maliha Khalid, Muhammad Usman, Muhammad Zawar Asif, Daphne Macbruce

**Affiliations:** 1 Internal Medicine, Mercy Hospital Fort Smith, Fort Smith, USA; 2 Internal Medicine, Jinnah Sindh Medical University, Karachi, PAK; 3 Internal Medicine, Arkansas College of Osteopathic Medicine, Fort Smith, USA; 4 Pulmonology and Critical Care, Mercy Hospital Fort Smith, Fort Smith, USA

**Keywords:** acute hypoxic respiratory failure, aspirin and ticagrelor (dapt), diffuse alveolar hemorrhage, diffuse interstitial lung disease, drug-related side effects and adverse reactions

## Abstract

Diffuse alveolar hemorrhage (DAH) is a rare and potentially life-threatening cause of acute hypoxemic respiratory failure and may be difficult to diagnose in patients with underlying interstitial lung disease (ILD). Ticagrelor frequently causes dyspnea, but DAH related to its use is exceedingly uncommon. We report the case of a 74-year-old man with chronic ILD and recent percutaneous coronary intervention who presented with progressive dyspnea, hypoxemia, and diffuse bilateral ground-glass opacities while receiving dual antiplatelet therapy with ticagrelor. Initial evaluation favored infection or ILD progression. Bronchoscopy with bronchoalveolar lavage demonstrated progressively bloody aliquots, establishing the diagnosis of DAH. Infectious, autoimmune, and thromboembolic causes were excluded, and the temporal relationship to ticagrelor supported a drug-induced etiology. This case highlights the diagnostic challenge of DAH in ILD patients and emphasizes the need for early consideration of medication-related hemorrhage in patients receiving ticagrelor who develop unexplained hypoxemia or hemoptysis.

## Introduction

Bleeding into the alveolar spaces of the lungs characterizes the syndrome of diffuse alveolar hemorrhage (DAH). Hemoptysis is usually the presenting symptom; however, it is not always present, even when hemorrhage is severe. DAH is a rare but life-threatening clinical condition that can rapidly progress to respiratory failure, often necessitating urgent mechanical ventilation and prompt management [1]. DAH is most frequently associated with autoimmune disorders, vasculitides, infections, and coagulopathies, although drug-induced cases have also been described. Dyspnea is a well-recognized adverse effect of ticagrelor and is reported in up to 20% of patients; however, DAH attributable to ticagrelor use is exceedingly rare, with only a few cases documented in the literature [2,3]. We present a case of ticagrelor-induced DAH in a patient with underlying interstitial lung disease (ILD), highlighting the diagnostic challenges and therapeutic considerations in this clinical scenario.

## Case presentation

A 74-year-old Caucasian male with a medical history significant for coronary artery disease (CAD) status post percutaneous coronary intervention (PCI) to the left anterior descending (LAD) artery two months prior, transcatheter aortic valve replacement (TAVR), hypertension, prediabetes, and chronic interstitial lung disease (ILD) of unclear etiology presented to the emergency department (ED) with progressive dyspnea and acute hypoxic respiratory failure.

Over the preceding month, the patient had been managed as an outpatient by pulmonology for worsening exertional dyspnea. Pulmonary function tests revealed no obstructive lung disease but mild diffusion capacity impairment, consistent with ILD. Serial high-resolution chest CT scans demonstrated bilateral reticular infiltrates and mediastinal and hilar lymphadenopathy without radiographic features of usual interstitial pneumonia (UIP), honeycombing, or traction bronchiectasis. His ILD had been clinically and radiographically stable and was managed conservatively. Approximately one month prior to presentation, home oxygen therapy was initiated due to worsening hypoxemia.

**Figure 1:**
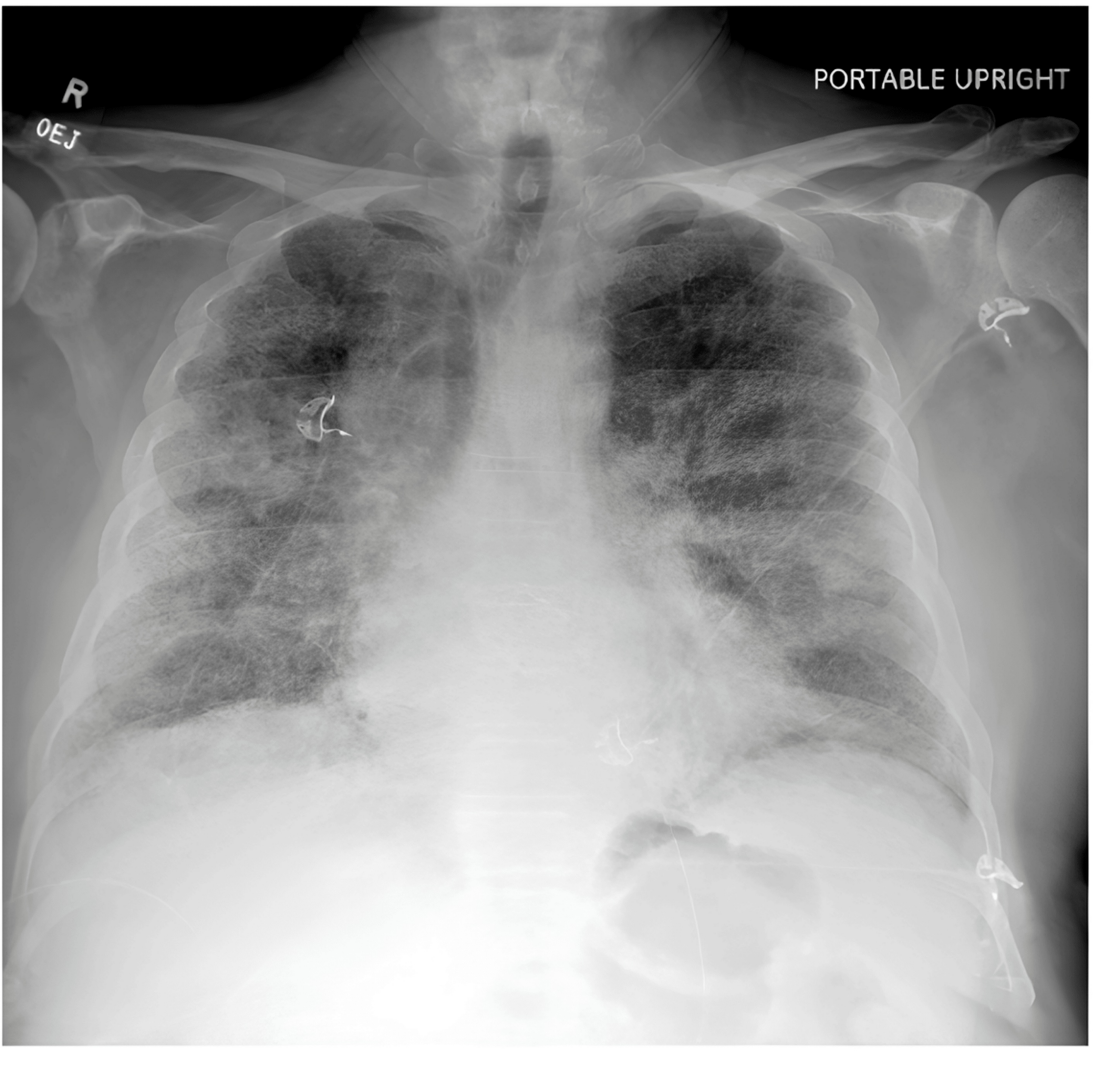
Chest radiograph demonstrating diffuse bilateral increased interstitial lung markings, most consistent with diffuse interstitial infiltrates

**Figure 2:**
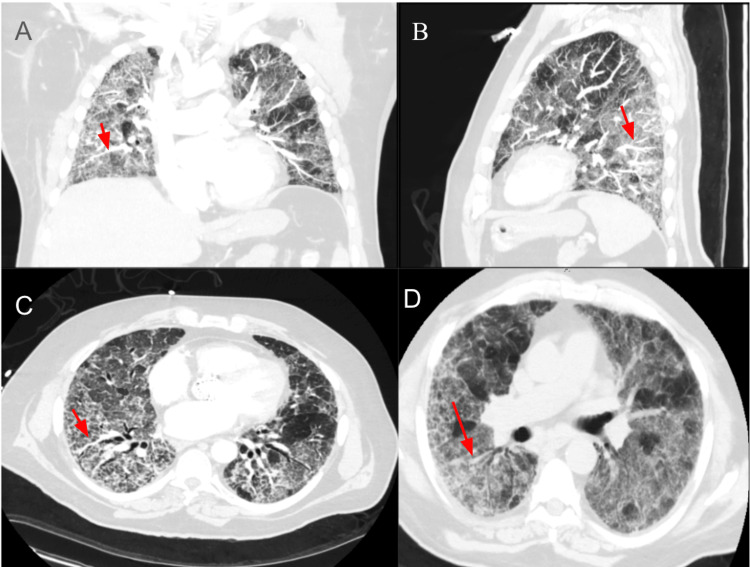
Computed tomography angiography (CTA) of the chest demonstrating diffuse interstitial lung disease (panels A, B, C, D), characterized by bilateral ground-glass opacities (red arrows in panels B & D) and interlobular septal thickening (red arrows in panels A & C). No evidence of honeycombing is observed.

One week prior to admission, he began experiencing increasing oxygen requirements and intermittent desaturation into the 70s on home oxygen. His family reported episodes of dusky appearance and labored breathing. He denied fever but endorsed subjective chills and mild, non-radiating chest discomfort described as fleeting muscle twinges. He denied palpitations, recent antibiotic use, or inhaler use. Notably, he reported a brief episode of mild hemoptysis that resolved spontaneously. Two months prior, the patient had undergone PCI with placement of a drug-eluting stent in the LAD and was started on dual antiplatelet therapy (DAPT) with ticagrelor and aspirin. Following the initiation of ticagrelor, his dyspnea progressively worsened. At ED presentation, he was hypoxic, with oxygen saturations in the 70s despite high-flow oxygen. He was tachypneic and in mild respiratory distress. Oxygenation failed to improve significantly despite escalation to high-flow nasal cannula (Airvo, Fisher & Paykel Healthcare Limited, Auckland, New Zealand) at 60 L/min with FiO₂ of 90-100%. Initial laboratory evaluation revealed leukocytosis (WBC 13,000/µL), negative pro-B-type natriuretic peptide (pro-BNP; 372pg/mL), and elevated C-reactive protein. Coagulation profile, including prothrombin time (PT)/international normalized ratio (INR) and activated partial thromboplastin time (aPTT), was in the normal range. Arterial blood gases revealed a P/F ratio (ratio of arterial oxygen partial pressure to inspired oxygen fraction) of 62, consistent with the development of severe respiratory distress. An echocardiogram revealed an ejection fraction of 65% with grade 1 diastolic dysfunction without any evidence of valvular or inferior vena cava abnormalities. Chest radiograph (Figure 1) and CT imaging demonstrated diffuse bilateral ground-glass opacities and interstitial infiltrates, raising concern for infectious pneumonia or pulmonary edema. CT pulmonary angiography was negative for pulmonary embolism but confirmed diffuse ILD with bilateral lower-lobe predominant interlobular septal and pleural thickening and mediastinal lymphadenopathy (Figure 2).

He was admitted with acute-on-chronic hypoxemic respiratory failure, presumed secondary to pneumonia and sepsis in the context of ILD. Empiric antibiotics (piperacillin-tazobactam and doxycycline) and intravenous fluids were initiated. Despite therapy, his respiratory status worsened rapidly, necessitating escalation to non-invasive positive pressure ventilation (NIPPV) and transfer to the intensive care unit (ICU).

**Figure 3:**
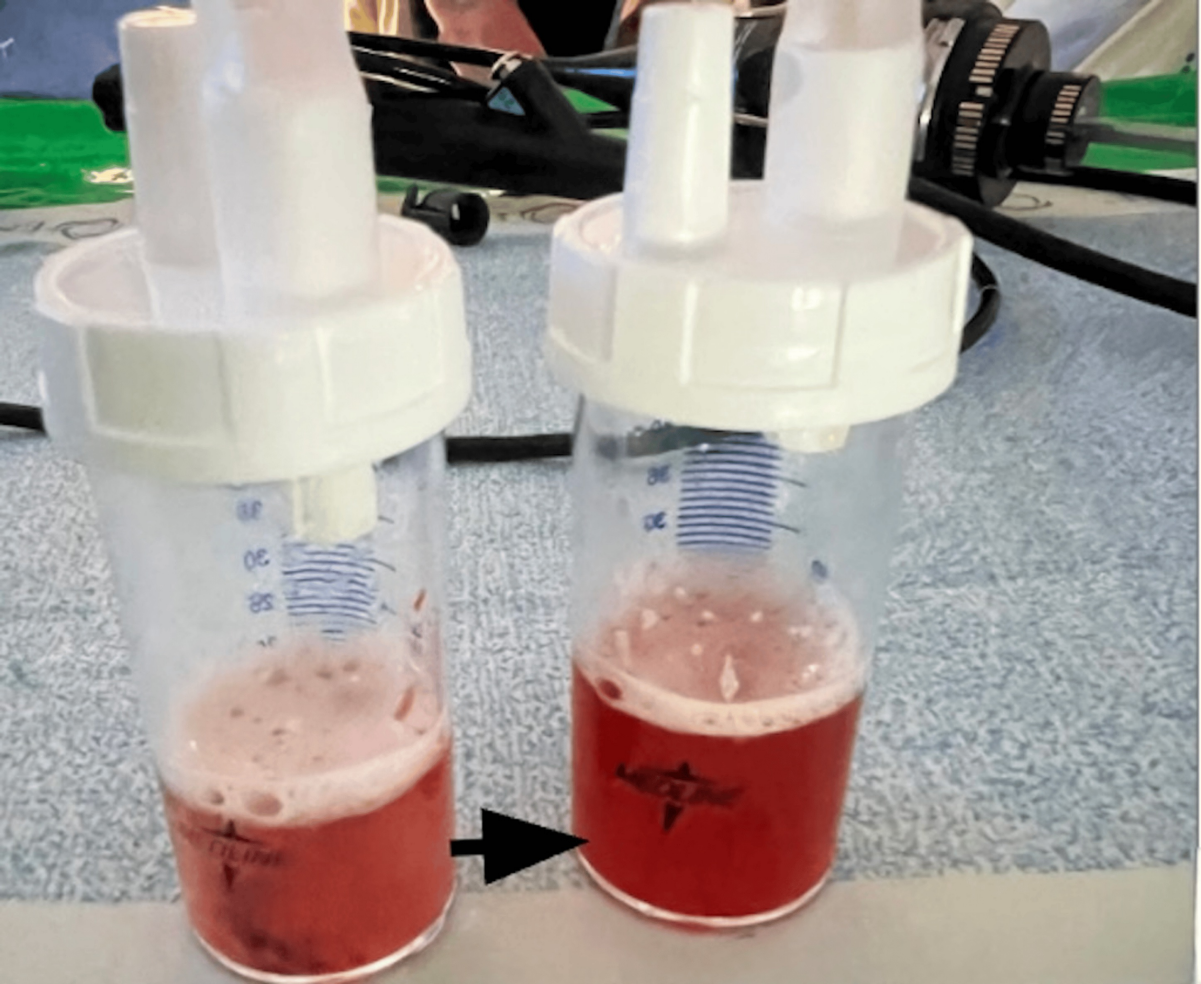
Sequential bronchoalveolar lavage (BAL) samples demonstrating progressively bloodier return (marked by arrowhead), a finding suggestive of diffuse alveolar hemorrhage

On hospital day three, persistent hypoxia and respiratory fatigue required endotracheal intubation and mechanical ventilation. Pulmonology was consulted due to the patient's prior ILD, history of hemoptysis, and diffuse infiltrates. Flexible bronchoscopy with bronchoalveolar lavage (BAL) revealed grossly bloody fluid with clots in both lower lobes, demonstrating bloody BAL output (Figure 3), consistent with diffuse alveolar hemorrhage (DAH).

No endobronchial masses or lesions were identified. BAL cultures were initially negative; fungal cultures grew Candida, considered to be colonization. An autoimmune panel was also negative for any underlying autoimmune etiology. Given the recent initiation of ticagrelor and the absence of other identifiable causes, ticagrelor-induced DAH was suspected. In consultation with cardiology, ticagrelor was discontinued, and antiplatelet therapy was modified to clopidogrel monotherapy, balancing the risks of hemorrhage with the need for antiplatelet therapy due to recent LAD stenting. The patient received systemic corticosteroids, lung-protective ventilation, and supportive care in the ICU. His course was complicated by hypotension requiring vasopressors, electrolyte disturbances, and metabolic acidosis. Procalcitonin remained low throughout; there was no pathological growth on BAL respiratory, fungal, and acid-fast bacilli cultures; and pneumonia pathogen PCR stayed negative, supporting a non-infectious etiology. Despite maximal therapy, respiratory failure persisted with no meaningful improvement in gas exchange or clinical status, with the patient persistently requiring 100% FiO₂. After nine days of mechanical ventilation, and following extensive multidisciplinary discussions, the patient's family opted for withdrawal of life-sustaining treatment in accordance with the patient's previously expressed wishes and poor prognosis. The patient was extubated and transitioned to comfort care on hospital day 10. He passed away peacefully shortly thereafter.

## Discussion

Our case describes a rapidly fatal DAH temporally associated with ticagrelor in an elderly man with chronic interstitial lung disease (ILD), prior TAVR, and recent PCI with a drug-eluting stent requiring DAPT. While DAH is an uncommon but catastrophic cause of respiratory failure, diagnosis is often delayed because its presentation with dyspnea and ground-glass opacities mimics infection, pulmonary edema, or ILD exacerbation [4,5].

What makes this report distinctive is the confluence of three high-risk features: advanced age with ILD (fragile alveolar-capillary interface), recent coronary stenting (stringent antiplatelet needs), and ticagrelor-associated bleeding physiology, culminating in fulminant DAH confirmed via bronchoscope. By tracing the reasoning behind each step, such as sepsis management, critical care escalation, bronchoscopy, antimicrobial choices, antiplatelet adjustment, and ventilation strategy, this case highlights the key decision points where earlier recognition of ticagrelor-related DAH might have changed the outcome. More broadly, it underscores that in hypoxemic ILD patients on DAPT, particularly ticagrelor, new or worsening dyspnea with hemoptysis should trigger early consideration of DAH and expedited bronchoscopy with medication withdrawal.

Previous reports of ticagrelor-related DAH are rare and often confounded by other comorbidities or anticoagulant use [2]. Unlike earlier cases, ours highlights how ILD amplifies bleeding susceptibility and masks recognition, delaying diagnosis until hypoxemia becomes refractory [4,6]. This underscores the need for early suspicion when ILD patients on DAPT, particularly ticagrelor, present with hemoptysis and diffuse infiltrates.

Ticagrelor, a potent reversible P2Y12 antagonist, produces stronger platelet inhibition than clopidogrel but at the cost of increased bleeding [7,8]. DAH in this context reflects impaired primary hemostasis rather than immune capillaritis [9]. Furthermore, ticagrelor elevates extracellular adenosine, inducing vasodilation and capillary leakage, potentially destabilizing already remodeled ILD vasculature [10]. Therefore, ILD-related capillary fragility, endotheliopathy of aging, and systemic platelet inhibition reasonably predisposed this patient to hemorrhage.

Early management was guideline-compliant: empiric antibiotics for potential pneumonia, fluid resuscitation for sepsis, and CT angiography to rule out embolism [11]. However, the hemoptysis, low procalcitonin, and diffuse ground-glass opacities should have raised suspicion earlier for bronchoscopy and ticagrelor discontinuation. BAL with increasingly bloody aliquots without an endobronchial source established the diagnosis of DAH [12].

A therapeutic dilemma existed between controlling the bleeding and avoiding stent thrombosis. Ticagrelor was stopped, and clopidogrel monotherapy was initiated, a pragmatic compromise given the recent LAD stent. Platelet transfusion is not effective in ticagrelor exposure because of residual drug effect, and a reversal agent (bentracimab) is still under investigation [13]. Adjunctive corticosteroids were administered, although their role in bland, drug-induced DAH remains uncertain [9]. Even with lung-protective ventilation, the hemorrhage was refractory, demonstrating the dismal prognosis when DAH superimposes on fibrotic ILD.

This case builds on previous accounts by highlighting how ILD predisposes patients to DAH while hindering earlier recognition through overlapping clinical and radiographic characteristics. Two subtle warning signs-hemoptysis and dyspnea within a few days of ticagrelor initiation-might have prompted earlier antiplatelet reevaluation. Embedding such red flags into post-PCI discharge education may shorten the time to intervention. Importantly, DAH checklists (recent antiplatelet initiation, hemoptysis, falling hemoglobin, diffuse ground-glass opacities, bloody BAL) can aid clinicians in early identification.

## Conclusions

In conclusion, ticagrelor-related DAH, though rare, can be fatal in elderly patients with ILD. Prompt recognition requires high clinical suspicion when hemoptysis and diffuse infiltrates occur in patients on DAPT. Early bronchoscopy and urgent antiplatelet modification are crucial. By highlighting the diagnostic pitfalls and the management tightrope between bleeding control and stent protection, this case contributes to the limited literature and may help clinicians intercept similar trajectories earlier.
